# Reconstruction of Respiratory Flow from an Impedance-Based Contactless Sensor System

**DOI:** 10.3390/s25237114

**Published:** 2025-11-21

**Authors:** Moritz Bednorz, Jan Ringkamp, Lara-Jasmin Behrend, Philipp Lebhardt, Jens Langejürgen

**Affiliations:** Fraunhofer Institute for Manufacturing Engineering and Automation IPA, Theodor-Kutzer-Ufer 1-3 (Cubex41), 68167 Mannheim, Germanyphilipp.lebhardt@ipa.fraunhofer.de (P.L.);

**Keywords:** contactless sensor, ISM radio band, machine learning, monitoring, non-invasive, respiratory signal detection, soft sensor

## Abstract

Conventional respiratory monitoring is often invasive, while most non-contact technologies like radar or cameras are limited to estimating respiratory rate, failing to reconstruct the detailed waveform of the respiratory flow itself. This gap limits their clinical utility for advanced diagnostics. We introduce a novel system that bridges this gap by combining a contactless, impedance-based sensor (the Thoraxmonitor) with a dedicated machine learning framework to directly reconstruct the full respiratory flow signal. Operating at 433 MHz, the system’s antenna array detects subtle changes in thoracic impedance, which are then translated into a quantitative flow signal by a Multilayer Perceptron Regressor. Based on data from 17 subjects benchmarked against a gold-standard flowmeter, our system accurately detected 98% of respiratory cycles. It achieved remarkable precision in timing respiratory events, with mean deviations of + 60 ms (±79 ms) for inspiration and + 50 ms (±63 ms) for expiration, making it suitable for time-critical applications. While a systematic bias in absolute tidal volume prediction currently limits inter-subject comparisons, the system excels at tracking relative intra-subject changes. Crucially, our model quantifies its own reliability, providing an intrinsic self-assessment mechanism. This work demonstrates a significant step beyond simple rate detection towards comprehensive, comfortable, and reliable respiratory analysis in clinical and everyday settings.

## 1. Introduction

The monitoring of respiratory patterns is critical in clinical care, yet current methods present a significant trade-off. Gold-standard spirometry is cumbersome and requires patient cooperation, whereas existing non-contact technologies are typically limited to measuring only the respiratory rate. They often fail to reconstruct the complete, quantitative waveform of the respiratory flow. The waveform encodes clinically relevant features such as the inspiratory-to-expiratory time ratio (I:E), the timing of inspiratory/expiratory onsets, peak inspiratory and expiratory flows as well as overall shape of the expiratory limb. Deviations in these features are characteristic of airway pathologies such as chronic obstructive pulmonary disease (COPD). Nevertheless, most non-contact technologies infer respiration indirectly from chest-wall motion and therefore recover only a surrogate of intrathoracic airflow.

This paper addresses this gap by presenting an integrated system that combines a contactless, impedance-based sensor, developed by some of the authors [1], with machine learning to predictively model the full respiratory flow waveform. The novelty of this work lies in the evaluation layer by reconstructing the full, quantitative respiratory flow waveform rather than detecting only the presence of respiration. It utilizes a 433 MHz antenna array to capture changes in the thoracic electrical impedance, leveraging the principle that air and pulmonary tissue exhibit different electrical properties, leading to measurable impedance changes during breathing [2,3]. To our knowledge, this study represents the first instance of employing regression models in conjunction with such a transmitting electromagnetic modality to quantitatively reconstruct the respiratory signal waveform. It should be noted that this work is presented as a first feasibility step to demonstrate the potential of this sensor technology for waveform reconstruction. Confirming generalizability and clinical validity with diagnostic intent will require larger, more diverse cohorts and prospective evaluations.

To position our contribution within the current landscape, Table 1 provides a systematic comparison of state-of-the-art non-contact respiratory monitoring technologies. Radar-based systems, including continuous wave (CW) and frequency-modulated continuous wave (FMCW) radars, are widely used due to their ability to capture subtle chest movements caused by breathing. These systems measure the Doppler shift in the reflected radio waves to estimate respiratory rates and patterns [4,5]. However, they are susceptible to motion artifacts, and the signal processing required to separate respiratory harmonics from the cardiac signal can be complex [5]. Optical systems provide another non-invasive means of monitoring respiration, primarily through video cameras or photoplethysmography (PPG). Video-based methods analyze thoracic movements using techniques like optical flow or motion factorization [6,7], whereas imaging PPG (iPPG) detects subtle changes in skin color caused by blood volume pulsations [8]. Although they are low-cost and widely accessible, these methods require a direct line-of-sight and are sensitive to changes in ambient lighting. Thermal imaging offers a more direct measure by detecting the temperature of airflow at the nostrils, but it suffers from a low signal-to-noise ratio and is sensitive to environmental conditions [7]. Other technologies measure changes in the body’s physical or electrical properties. Capacitive and inductive sensors, for example, detect variations in the thoracic electric or magnetic fields caused by chest movements and can be integrated into furniture or textiles, though they require close proximity [9]. Ultrasound sensors measure the Doppler shift in exhaled air, offering a direct airflow measure, but they are sensitive to air currents and have a limited range [10]. Laser Doppler Vibrometers (LDVs) provide a high-precision alternative, capable of capturing minute skin vibrations from cardiorespiratory activity by measuring the Doppler shift in a reflected laser beam [11,12,13]. Ballistocardiography (BCG) operates on a mechanical principle, measuring the body’s recoil forces in response to cardiac blood ejection, which can be captured unobtrusively by sensors in beds or chairs but is highly prone to motion artifacts [14]. Furthermore, research has been conducted into transmission modalities as alternatives to the reflective techniques employed in radar systems or LDV. For instance, Uysal et al. [15] have described systems comprising a separate transmitter and receiver, where the subject is positioned in the signal propagation path. These methods detect the modulation of the transmitted signal caused by the external thoracic movement.

This synergistic approach of our impedance-based sensor and machine learning moves beyond simple rate detection to enable a more detailed respiratory analysis. To substantiate the claims made, this paper is structured as follows: we provide (1) the validation of the impedance-based sensor system against a gold-standard flowmeter across a diverse subject cohort; (2) a detailed performance evaluation of machine learning techniques for signal reconstruction; and (3) a thorough analysis of the system’s accuracy in deriving advanced respiratory parameters.

## 2. Materials and Methods

### 2.1. Thoraxmonitor

**Figure 1:**
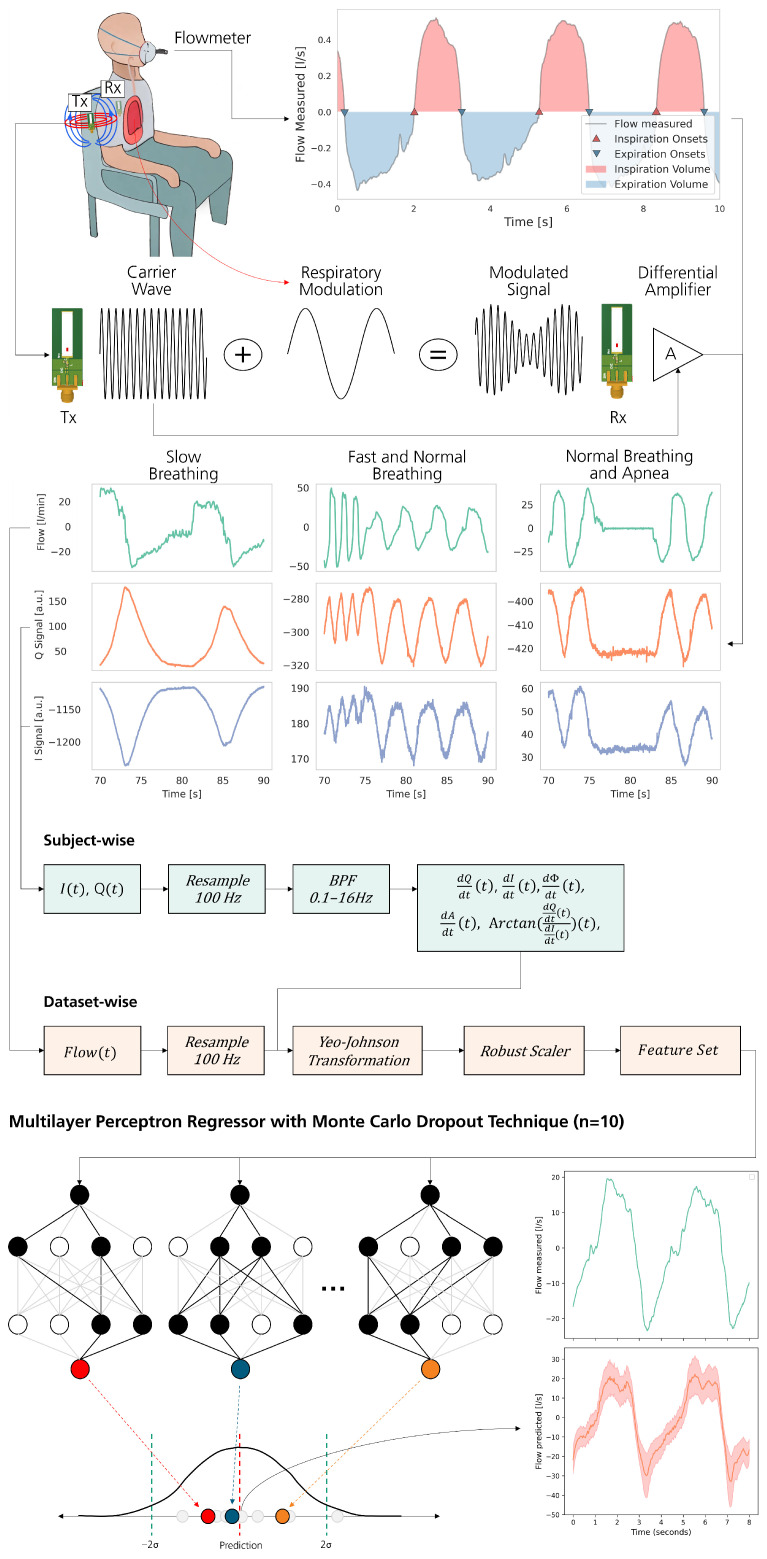
Illustration of the experimental setup and signal processing pipeline. The top left image shows the subject seated with the contactless sensor system, capturing respiratory flow using a 433 MHz antenna array whereas simultaneously measuring the flow signal as a reference. The top right plot displays the flow signal, highlighting inspiration and expiration phases, as well as their onsets. The next section illustrates the process of modulated carrier wave demodulation to extract phase information. Subsequent plots present the raw flow and Q/I signals. The following section outlines the preprocessing pipeline applied to each subject or dataset, including resampling, bandpass filtering, transformation, and feature extraction. The final plot depicts the Monte Carlo process using a Multilayer Perceptron, showing the reconstructed flow signal along with reliability intervals.

The Thoraxmonitor is a contactless sensor system designed to measure respiratory patterns by detecting changes in the thoracic electrical properties. Its core electronics are directly connected via coaxial cables to a pair of ultra high frequency (UHF) antennas operating at 433 MHz. These antennas are coupled through their mutual impedance to detect a variation between the emitted and received signals, as shown in Figure 1. This near-field impedance-based approach was chosen as it is highly sensitive to dielectric changes within the entire thoracic cavity caused by air filling the lungs, providing a volumetric signal that is more directly related to respiratory flow than surface-motion measurements from radar or optical systems.

The physical principle of the measurement is based on the mutual impedance ($Z_{12}$) between the two antennas. For two small electric dipoles, this can be modeled as [16]:

(1) $$Z_{12}=\frac{l^{2}}{4\pi r^{2}}\left[\underset{C}{\underset{︸}{\frac{1}{j\omega \epsilon r}}}+R_{0}+\underset{L}{\underset{︸}{j\omega \mu r}}\right]e^{-j2\pi r/\lambda },$$

where *l* is the dipole length, *r* is the distance between the dipoles, ϵ is the electric permittivity of the medium, and $R_{0}$ is the wave resistance in free space. The term in the brackets can be represented by an equivalent circuit consisting of a capacitive (C), resistive ($R_{0}$), and inductive (L) component. Under the near-field conditions of our application, the capacitive term, which is inversely proportional to the permittivity ϵ, dominates the mutual impedance.

During respiration, the lungs fill with air, causing a significant change in the overall permittivity of the thoracic cavity [2,3,17]. This change in ϵ directly modulates the mutual impedance $Z_{12}$, resulting in a measurable shift in the amplitude and phase of the received signal. Although an I/Q demodulation is used to quantify these variations, a method common in RADAR applications, this physical model confirms that the measurement effect is based on changes in the dielectric properties of the medium. Surface effects, such as a Doppler shift in a reflected wave, do not play a role in this consideration.

#### 2.1.1. Data Acquisition

**Figure 2:**
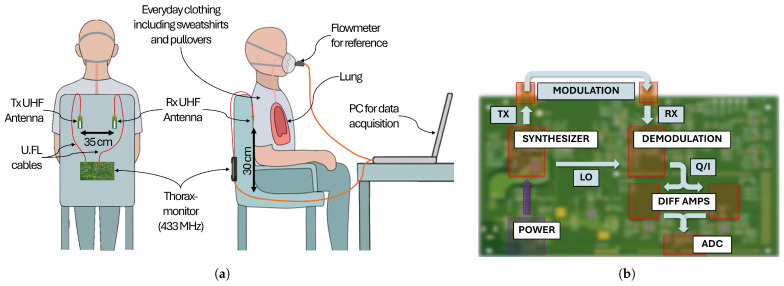
Data acquisition workflow combining the Thoraxmonitor and a flowmeter. (**a**) The experimental setup with the Thoraxmonitor antennas and the concurrent reference flowmeter recording, highlighting the relative sensor placement and subject posture. The Thoraxmonitor electronics are mounted on the rear of the chair and connected via U.FL cables to a transmit (Tx) and receive (Rx) UHF antenna pair separated by 35 cm. The antennas are fixed 30 cm above the seat level to couple through the thoracic region while the participant remains in everyday clothing. A nose mask with an inline flowmeter provides the reference flow signal, which, together with the Thoraxmonitor data, is recorded on a laptop positioned in front of the subject. (**b**) The electronics chain from the 433 MHz synthesizer to the digitization stage, illustrating the signal processing path that yields the in-phase and quadrature data used throughout this work. A synthesizer (LMX2571) generates the 433 MHz carrier signal, which is transmitted via the TX antenna. The signal received at the RX antenna, modulated by thoracic impedance changes, is processed by an I/Q demodulator (LTC5585). The resulting baseband signals are amplified by differential amplifiers (OPA2316ID) and digitized by the Analog-to-Digital Converter (ADC) of an Arduino Due for further processing.

The data acquisition setup, modified from Ringkamp et al. [1], consists of the Thoraxmonitor affixed to a chair, featuring the core evaluation unit (Figure 2b) on the back of the chair backrest and two coupled UHF antennas attached to the front of the chair backrest, operating at a frequency of 433 MHz, as illustrated in Figure 2a. Data acquisition was facilitated through the use of coupled multilayer ceramic chip antennas, which were integrated onto custom printed circuit boards (PCBs), positioned 30 cm above the chair’s seating area and spaced 35 cm apart. The antennas PCBs were connected to the electronic module with U.FL cables of 1 m length. The transmitter power was at about 470 μW. The experiments involved measuring respiratory signals in a sample of 17 healthy individuals, aged 20 to 40 years, over a duration of nine months. Informed consent was obtained from all subjects involved in the study. The subjects wore everyday clothing, including sweatshirts and pullovers. During the recordings, they received instructions to perform normal, fast, and slow breathing patterns.

The experiment recorded In-Phase (I) and Quadrature (Q) signals from the continuous wave (CW) sensor system, with simultaneous tracking of the respiratory signal using a nose mask connected to a flowmeter (Sensirion SFM3000, Sensirion AG, Stäfa, Switzerland) operating at a sampling rate of approximately 100 Hz. The flowmeter is oriented in a way that inhalation produces positive signals. A microcontroller digitizes the I/Q signals with a sampling rate of 33 Hz. To synchronize the timestamps from the flowmeter and the Thoraxmonitor, a common time window was identified by finding the maximum start time and minimum end time of the signals. The Thoraxmonitor’s time grid served as the reference, and the flowmeter data was linearly interpolated onto each Thoraxmonitor timestamp.

#### 2.1.2. Data Wrangling

Initial preprocessing involved resampling the I/Q signals to 100 Hz using linear interpolation and deriving their phase and amplitude components. Calibration of these signals is necessary due to the prevalent imperfections inherent to I/Q signals within practical hardware frameworks. Optimally calibrated I/Q signals should manifest an arc trajectory within the complex plane, characterized by a circular pattern centered at the origin. Addressing this, the process incorporates the application of a third-order Butterworth filter with a bandpass frequency range of 0.1 Hz to 16 Hz as suggested by Wu et al. [18], aimed at eliminating extraneous non-respiratory signal elements. Afterwards the derivatives for each signal are computed on an individual subject basis. To standardize the dataset across the participant cohort, Yeo-Johnson Power Transformations and Robust Scaling are applied dataset-wide, encompassing all subjects. These transformations are similarly applied to the respiratory signals, ensuring consistent preprocessing across all data modalities. Figure 1 outlines the preprocessing pipeline.

This preprocessing yielded five derived features:

- Time derivatives of *Q* and *I* signals;
- Time derivative of amplitude and phase;
- A phase-related feature calculated from the derivatives of *Q* and *I*;
- The target respiratory signal.

#### 2.1.3. Model Selection

We employed the Automated Machine Learning framework Lazypredict (https://lazypredict.readthedocs.io/en/latest/, accessed on 5 May 2025) to evaluate 32 scikit-learn regression algorithms using default configurations. This mitigated confirmation bias and adhered to the “No Free Lunch” theorem, positing no universal superiority among optimization algorithms [19]. To ensure a subject-independent evaluation and prevent optimistic performance estimates, we employed a Group-10-fold cross-validation scheme. The data was partitioned into groups based on subject ID, ensuring that all data from a single subject were exclusively in either the training or the validation set within each fold. The OPTUNA framework (https://optuna.org/, accessed on 28 May 2025), through 100 iterations of Group-5-Fold validation, optimized hyperparameters. To assess the reliability of the model’s predictions, we used Monte-Carlo Dropout techniques to generate a measure of prediction confidence, with ten predictions under slight variations as demonstrated in Figure 1. The mean and standard deviation of these predictions established a confidence threshold at a variable value, dependent on the desired balance between sensitivity and specificity. In our case, we assumed a balanced confidence value at 50%. Although this approach does not provide formally validated, calibrated probabilistic uncertainty, it serves as a practical tool for flagging predictions whose results are unreliable, thereby increasing the overall reliability of the system output.

#### 2.1.4. Performance Evaluation

The primary evaluation metric within the Automated Machine Learning pipeline was the Median Absolute Error, chosen for robustness against outliers. Subsequent analyses with the best-performing model, included comparisons of predicted respiratory metrics such as:

- Count of respiratory cycles;
- Duration of each respiratory cycle;
- Tidal volume of each respiratory cycle.

These evaluations provided insights into the model’s precision and reliability across various respiratory conditions and subjects.

## 3. Results and Discussion

### 3.1. Dataset Overview

#### 3.1.1. Data Selection and Quality Assessment

To ensure data quality, all recordings were screened based on two predefined exclusion criteria. First, recordings containing significant motion artifacts, identified through visual inspection by the research team, were discarded. Second, signals with a signal-to-noise ratio (SNR) below 10 dB were excluded to guarantee a minimum level of signal integrity. Applying these criteria resulted in the exclusion of data from two subjects. The final dataset used for analysis comprises 97.35 min of recordings, spanning 1442 breathing cycles.

#### 3.1.2. Classification of Respiratory Patterns

The collected breathing cycles were classified into four distinct patterns based on their temporal characteristics, following established clinical definitions [20]. The distribution of these patterns in our dataset was as follows:

- Fast Breathing (≥20 breaths per minute; cycle duration of ≤ 3 s): ≈20% of cycles.
- Normal Breathing (10–20 breaths per minute; cycle duration between 3 s to 6 s): ≈48% of cycles.
- Slow Breathing (≤10 breaths per minute; cycle duration ≥ 6 s): ≈20% of cycles.
- Apnea (absence of breathing): ≈12% of cycles.

This categorization highlights the diverse range of respiratory dynamics captured in the study, providing a robust basis for model evaluation across different breathing rates.

#### 3.1.3. Feature Extraction and Signal Analysis

To evaluate the model’s performance, we extracted critical features from both the reference and the predicted respiratory flow signals. These features included the onset points of inhalation and exhalation, and the tidal volume for each respiratory cycle, as illustrated in Figure 1. Onset points were identified by detecting zero-crossings in the respiratory signal. The duration of each cycle was determined by measuring the time differences between these points. Tidal volume was calculated by integrating the respiratory signal over time for each cycle detected. Analysis of the reference sensor data revealed that the duration of breathing cycles predominantly ranged from 3 s to 6 s, consistent with normal respiratory rates [20]. Tidal volumes measured by the reference sensor were most frequently recorded in the 0.5 L to 0.6 L range.

#### 3.1.4. Signal Integrity and Correlation Analysis

The integrity of the I/Q signal, a pivotal aspect for accurate respiratory measurements. I/Q signals from all 15 valid participants exhibits a uniform orientation and morphology across the dataset, with data clusters displaying a nearly tangential orientation relative to the center in complex Q/I domain, indicating a consistent signal profile among the subjects. Furthermore, a comparative analysis of raw and preprocessed I/Q signals in relation to the respiratory signal revealed a correlation between the I and Q signals, attributable to their common source as well as their correlation to the respiratory flow. The analysis demonstrated a correlation with the respiratory flow of 0.59 for the I signal and 0.57 for Q signal. This underscores the connection between sensor outputs and pulmonary volume, effectively serving as an integrative measure of the respiratory signal. These findings align with previous studies by Ringkamp et al. [1], reinforcing the validity of the sensor system. Preprocessing steps further enhanced this correlation. By replacing the raw I/Q signals with their first derivatives, the analysis established a stronger correlation with the respiratory signal. Mathematically, the raw I/Q signals I(t) and Q(t) reflect the instantaneous state of the thoracic impedance, which is influenced by the volume of air and tissue within the thorax at any given time. The first derivatives of these signals, $\frac{dI}{dt}\left(t\right)$ and $\frac{dQ}{dt}\left(t\right)$, represent the rate of change in impedance over time. Since the sensor system effectively measures the volume of air and tissue, the first derivative corresponds to the rate of change in this volume. This rate of change is directly related to the flow of air, as flow is defined as the change in volume over time. Therefore, the first derivatives $\frac{dI}{dt}\left(t\right)$ and $\frac{dQ}{dt}\left(t\right)$ have a higher correlation with the respiratory flow signal.

### 3.2. Automated Machine Learning

The evaluation of various machine learning models using the Automated Machine Learning framework Lazypredict identified the Multilayer Perceptron (MLP) Regressor as the optimal model for respiratory signal prediction in this context. The MLP regressor achieved a Median Absolute Error of 0.28, demonstrating superior predictive accuracy compared to other algorithms. The observed performance of the MLP, along with algorithms like Gradient Boosting, emphasizes the presence of non-linear relationships within the respiratory data, necessitating complex models for accurate prediction. Following the identification of the MLP regressor as the leading model, extensive hyperparameter tuning was conducted, resulting in an optimized MLP configuration with the following hyperparameters:

- Neuron configuration: [150, 550, 800, 200, 550];
- Learning rate: 0.00015;
- Dropout rate: 0.3 for regularization;
- Optimizer: AdamW;
- Loss function: HuberLoss.

The resulting architecture, was selected through a data-driven hyperparameter search, suggesting its capacity was appropriate for modeling the complexities in the data. Overfitting was explicitly addressed through the subject-independent cross-validation scheme, the use of a dropout rate of 0.3 for regularization, and the choice of the robust Huber loss function.

### 3.3. Accuracy of Signal Reconstruction

**Figure 3:**
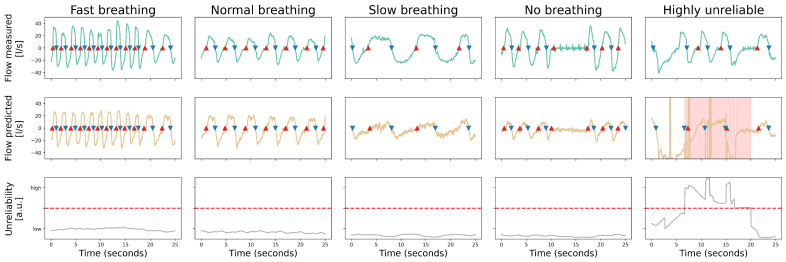
Series of graphical illustrations categorizing respiratory conditions and depicting the model’s performance. For each category, the figure shows the annotated reference sensor flow (green line), the predicted flow (orange line), the reliability score (grey line), and the reliability threshold (red dashed line). Intervals where the reliability score exceeds the threshold are highlighted with a red background in the predicted flow plot. Both, the reference and predicted flow are annotated with the onset of inspiration (red triangle) and expiration (blue triangle), calculated as described in Section 3.1.3.

The following results evaluate the performance of our integrated system in reconstructing the respiratory waveform. We analyze its accuracy in different breathing scenarios and introduce the model’s ability to quantify its reliability, a key feature for clinical confidence. Figure 3 comprises a suite of chronological graphical illustrations partitioned into five distinct respiratory categories: fast, normal, slow, no respiration and a highly unreliable. Each category is segmented into three diagrams, each serving a specific purpose:

- The upper diagram shows a quantification of the observed respiratory signal (in $L\;s^{-1}$) via the reference flow meter.
- The middle diagram shows the reconstructed respiratory signal for the corresponding duration. An area is marked in red to indicate the increased unreliability.
- The lower diagram illustrates the reliability score over time, with a dashed line indicating the threshold value above which the outcome is considered unreliable.

When examining fast breathing sequences, the measured respiratory signal shows a fast oscillating pattern. The reconstructed respiratory signal replicates this with a noticeable decrease in the tidal volume whereas preserving the natural frequency. The degree of unreliability remains below the threshold value, but is significantly higher in the areas where the amplitude difference in the signal has peak values. A similar trend is observed in standard breathing, slow breathing and no breathing, where the onset time of breathing cycles is determined with high accuracy, yet the respiratory signal amplitude is consistently underestimated. It is also noteworthy that the reconstructed signal-to-noise ratio deteriorates as the breathing rate decreases.

In the highly unreliable signal section, which represents a mixture of normal and apneic breathing, the strength of the reliability score becomes particularly evident. Whereas the model attempts to identify inspiration and expiration events, the associated predictions exhibit a very high level of unreliability, significantly exceeding the predefined threshold. This high score correctly flags these detected events as unreliable, leading to their effective rejection. In this case we observed a significant decrease in received 433 MHz signal, therefore a lack of information, possibly caused by a misplacement of the subject relative to the sensor array. By using a reliability threshold derived from 10 Monte Carlo predictions to reject unreliable detections, the model correctly identified 98% of respiratory cycles. These results highlight that whereas the model excels at detecting the presence and frequency of breathing cycles, its fidelity decreases when signal amplitude is a critical factor. Finally, the integration of a reliability score serves as a self-assessment mechanism. It provides a quantitative basis for accepting or rejecting the model’s output, thereby ensuring that the system is not only predictive but also aware of its own limitations.

### 3.4. Results Across Subjects

#### 3.4.1. Breathing Cycles

**Figure 4:**
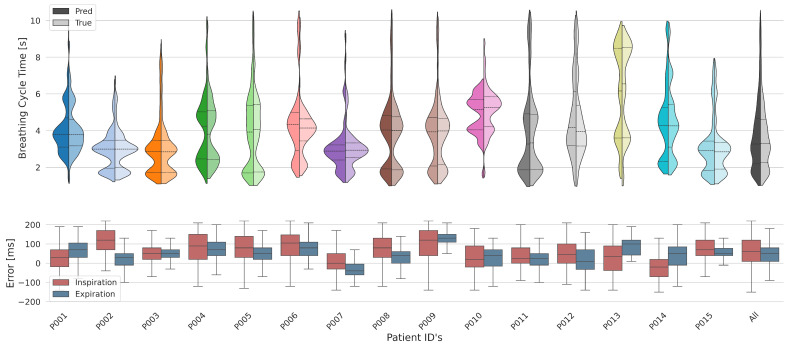
Violin plots comparing the duration of respiratory cycles measured and predicted across all subjects and the corresponding errors as a boxplot below. These plots reveal the variability in breathing patterns and the model’s capability to accurately predict respiratory cycle durations.

The violin plots in Figure 4 illustrate the distribution of breathing cycle times across all 15 subjects, comparing the measured and model-predicted durations. The mostly bimodal distribution within individual violins reflects variability in each subject’s breathing pattern.

The observed data (’True’) and the predictions (’Pred’) are in close agreement for most subjects, suggesting a model with good predictive performance. Notably, certain individuals exhibit a wider range in cycle times (e.g., P008), whereas others demonstrate more consistent cycle times (e.g., P002). The aggregated data (’All’) indicate a median cycle time that falls within the normal breathing range, with tails extending into the rapid and slow breathing domains. This overall pattern underlines the model’s capacity to encompass the diverse breathing rates present in the general population. This is evidenced by the model’s precision in timing, with a mean deviation in the onset of inspiration and expiration recorded at + 60 ms (± 79 ms) and + 50 ms (± 63 ms), respectively, as summarized in Table 2. These deviation values should be interpreted in the context of the system’s inherent limitations. The combination of the Thoraxmonitor’s 33 Hz sampling rate and non-synchronous data acquisition with the reference device imposes a theoretical floor on timing accuracy of approximately 30 ms, indicating that the model performs near the physical limits of the setup. However, it is noteworthy that next-generation versions of the sensor system support sampling rates over 1000 Hz. Furthermore, this timing accuracy was found to be independent of the breathing rate category, holding consistent across rapid, normal, and slow breathing patterns. In summary, the model demonstrates robustness in predicting breathing cycle times across varied respiratory rates. However, the extent of discrepancies between measured and predicted values for certain subjects may signal the need for further model refinement or indicate patient-specific factors that could be influencing breathing patterns.

#### 3.4.2. Tidal Volume

**Figure 5:**
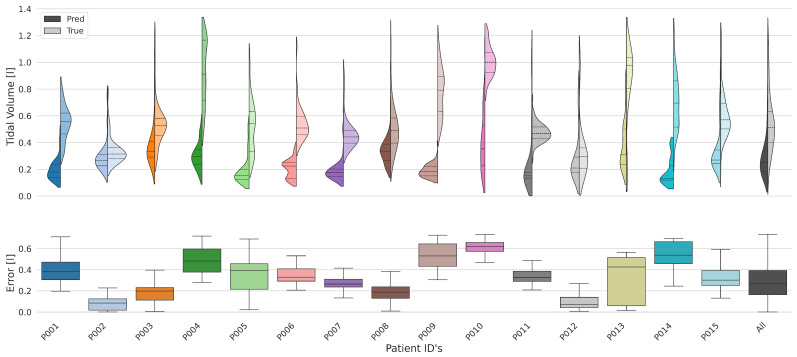
Comparative analysis of measured and predicted tidal volumes in different subjects, represented by violin plots. The plots illustrate the performance of the model in estimating tidal volumes and show a systematic bias in the predictions.

The analysis of tidal volumes, which represent the amount of air inhaled and exhaled during normal breathing, includes a detailed comparison between measured and predicted values for all participants. This comparison is visualized by violin plots, as shown in Figure 5, in which the actual measurements (“True”) are compared to the predictions of the model (“Pred”) for each subject.

The aggregated violin plot, which summarizes the data for the entire cohort, reveals a skewed normal distribution and provides an overall assessment of model performance. In the participant-specific plots, however, significant variability in predictive accuracy indicates a subject-dependent bias, as detailed in Table 3. This finding is attributable to minor differences in each subject’s posture and position relative to the sensor array. Crucially, whereas this inter-subject bias limits the comparison of absolute tidal volumes between individuals, it does not impede the system’s utility for intra-subject trend monitoring. For instance, the system could effectively track relative changes in a patient’s tidal volume following the administration of a bronchodilatory medication, which typically leads to an increase in tidal volume and higher respiratory flows [21,22].

**Figure 6:**
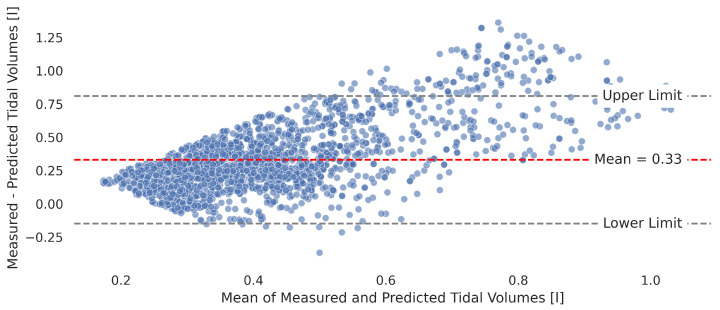
Bland–Altman plot showcasing the agreement between measured and predicted tidal volumes, identifying a systematic underestimation bias.

A subsequent analysis, particularly using the Bland–Altman diagram in Figure 6, illustrates the systematic underestimation of tidal volumes by the model, as evidenced by a central bias of 330 mL. This graph also shows a trend where the discrepancy between predicted and actual measurements increases with an increase in average tidal volume—a phenomenon known as heteroscedasticity. This pattern points to the difficulties of the model in accurately predicting larger tidal volumes and indicates a larger discrepancy at higher tidal volumes. Importantly, this amplitude-dependent error was found to be independent of the respiratory rate. This is consistent with the observation that, within our dataset, the subjects’ tidal volumes did not systematically vary with their breathing frequency, meaning the underestimation bias does not significantly change across fast, normal, or slow breathing cycles.

The primary cause of this bias is the loss of information regarding the shifted resonance frequency due to dielectric properties in the near field within the I and Q data received from the Thoraxmonitor. This information is crucial for accurately reconstructing the amplitude of individual subjects’ tidal volumes. Despite these issues, the model shows a good correlation with the respiratory flow, with a Pearson correlation of 0.67, even though it has a mean error of 267 mL (± 172 mL) in estimating tidal volume. However, the observed discrepancies and trends in systematic error emphasize the need for further refinements. Such improvements are essential to increase the precision of the model, especially when fitting predictions to individual respiratory functions.

## 4. Conclusions

In this paper, we presented an advanced non-contact impedance-based sensor system, the Thoraxmonitor, designed for the accurate monitoring of respiratory patterns. Utilizing a 433 MHz antenna array, the investigated contactless system captures thoracic impedance changes associated with respiratory cycles. The combination of this sensor system with an Automated Machine Learning framework significantly enhances the accuracy and reliability of respiratory signal detection and analysis. Our findings demonstrate the system’s robustness and efficacy across a diverse, but relatively small, cohort, successfully correlating sensor output with standard respiratory measurements. The application of machine learning techniques, particularly the use of the MLP regressor, enabled precise reconstruction of respiratory signals and the derivation of critical respiratory parameters such as inhalation/exhalation durations and onsets. The model achieved a detection rate of 98% for respiratory cycles, despite showing some decrease in tidal volume. Notably, the model’s predictions for the onset of inspiration and expiration deviate by + 60 ms (± 79 ms) and + 50 ms (± 63 ms), respectively, suggesting its potential utility in controlling respirators. A positive deviation indicates a delay in prediction. Furthermore, performance evaluation revealed a systematic bias in tidal volume predictions. This bias is primarily caused by the loss of information regarding the shifted resonance frequency due to dielectric properties in the near field within the I and Q data received from the Thoraxmonitor. This information is crucial for accurately reconstructing the amplitude of individual subjects’ tidal volumes. With a mean error of 267 mL (± 172 mL), the system’s estimation of absolute tidal volume is currently insufficient for diagnostic quantification in a clinical context without individual calibration. Such a calibration could be achieved by having the subject take a few controlled breaths while being measured by both the Thoraxmonitor and a reference flowmeter. The paired data could then be used to calculate a subject-specific scaling factor or a simple linear regression model to map the sensor’s output to absolute tidal volumes. This one-time procedure would enable accurate, quantitative measurements for the remainder of the session, assuming that the subject’s posture remains constant. Its value, however, lies not in absolute accuracy but in its ability to faithfully track relative intra-subject changes. This makes it a promising tool for monitoring trends, where the direction and magnitude of change are more critical than the precise baseline value, for instance in pharmacology for assessing a patient’s response over hours or days to respiratory medications such as bronchodilators.

The primary contribution of this research is to address a critical gap in respiratory monitoring. Conventional methods are often invasive or uncomfortable, whereas existing non-contact technologies like radar or cameras are typically limited to detecting only the respiratory rate. Our work demonstrates that a non-contact, impedance-based system can predictively model the full respiratory flow waveform. This reconstruction of the continuous signal is a significant advancement, as it enables the derivation of advanced, clinically relevant parameters, including highly accurate inhalation/exhalation onsets and durations. Whereas the prediction of absolute tidal volume is currently limited by a subject-dependent bias, the reconstructed waveform enables the monitoring of intra-subject trends. Crucially, the model also quantifies its reliability, providing a score for each derived data point. This self-assessment capability distinguishes robust measurements from noise and is a critical feature for building trust in clinical decision-making, a dimension entirely absent in simpler non-contact methods. By validating this approach against a gold-standard flowmeter and providing a detailed performance evaluation, this study highlights the potential of our system for accurate and comfortable respiratory monitoring in both clinical and everyday settings.

A key limitation of the current study is its relatively small and homogeneous cohort of 15 healthy, young subjects. Such a sample size may lead to an overestimation of performance and increases the risk of the model overfitting to cohort-specific characteristics, thereby limiting the generalizability of our findings. While the results successfully demonstrate the feasibility of the sensor technology, establishing clinical validity requires future studies with larger, more diverse cohorts that better represent the general population. Future research will therefore also focus on refining the sensor system and machine learning models to mitigate the identified biases and improve accuracy. This will involve capturing more comprehensive data on the shifted resonance frequency to reconstruct tidal volumes more accurately. Additionally, exploring more advanced signal processing and machine learning techniques, such as Long Short-Term Memory networks and transformer models, will be essential for enhancing the system’s applicability and reliability in diverse real-world scenarios. Data augmentation techniques are expected to improve the robustness of these predictions, whereas the integration of an extensive dataset will further refine the accuracy and reliability of predictive models. Real-life validation of these models, simulating real-time conditions, is crucial for verifying their practical applicability in clinical settings. Whereas current models efficiently predict standard respiratory patterns, the identification and interpretation of atypical, potentially pathological respiratory behaviors remain a challenge. Employing anomaly detection methods within machine learning could significantly contribute to differentiating and understanding such deviations, thereby augmenting the diagnostic utility of clinical decision-support systems.

## Figures and Tables

**Table 1:** Comparison of Non-Contact Respiratory Monitoring Technologies.

Technology	Measurement Principle	Primary Output	Advantages	Limitations
Radar [4,5]	Doppler shift in reflected radio waves from chest wall movement	Chest displacement, Rate	High sensitivity, Robust to lighting conditions, Can work through clothing	Susceptible to motion artifacts, Indirect flow measure, Complex signal processing for harmonics
Optical [6,7,8]	Image motion analysis (Optical Flow)/Light absorption changes (Imaging PPG)	Chest displacement, Rate, Blood volume pulse	Low cost, Widely available cameras, Can monitor multiple subjects	Line-of-sight required, Sensitive to lighting changes and shadows, Motion artifacts
Thermal Imaging [7]	Temperature difference between inhaled/exhaled air and ambient air	Airflow detection, Rate	Direct visualization of airflow at nostrils/mouth	Low signal-to-noise ratio, Highly sensitive to ambient temperature and air currents
Capacitive/Inductive [9]	Changes in electric field (capacitive) or magnetic field (inductive) due to thoracic impedance changes	Chest circumference/ volume change	Can be integrated into furniture (chairs, beds) or textiles	Requires close proximity, Signal quality depends on posture and position, Calibration needed
Ultrasound [10]	Doppler shift in ultrasonic waves reflected by exhaled air particles	Airflow detection, Rate	Non-ionizing, Direct airflow measure	Sensitive to ambient air currents, Limited range, Perturbed by ambient noise
Laser Doppler Vibrometry [11,12,13]	Doppler shift in laser light scattered from the skin surface	Skin surface velocity/ displacement, Rate	High precision and metrological quality, Can capture fine cardiorespiratory movements	Requires line-of-sight, Can be expensive, Sensitive to gross body movements
Ballisto-cardiography [14]	Measurement of body micromovements and recoil forces from cardiac blood ejection	Body acceleration/ displacement, Rate	Unobtrusive, Can be integrated into beds, chairs, or scales	Highly susceptible to motion artifacts, Indirect measure of respiration (often coupled with cardiac signal)
RF Sensing [15]	Modulation of the line-of-sight signal path by external thoracic movement	Chest displacement, Rate	Simple architecture (Tx/Rx), Can distinguish multiple subjects	Highly sensitive to any motion in the signal path, Environment-dependent
This Work	Transmission through the thorax to measure internal dielectric properties	Intrathoracic Air Volume Waveform	Direct volumetric signal for flow reconstruction	Subject position bias

**Table 2:** Subject-specific onset timing errors for inspiration and expiration. Positive median values indicate that the model predicts onsets later than the reference measurement. The aggregated row (’All’) represents the combined performance across all 15 subjects.

Subject ID	Inspiration Error (ms)		Expiration Error (ms)	
	median	std	median	std
All	60.0	±79.4	50.0	±62.6
P001	30.0	±80.5	70.0	±62.6
P002	120.0	±63.1	30.0	±49.0
P003	50.0	±57.0	50.0	±37.3
P004	90.0	±90.7	70.0	±76.7
P005	80.0	±85.6	50.0	±59.7
P006	105.0	±83.5	80.0	±48.8
P007	0.0	±67.5	−40.0	±50.2
P008	80.0	±76.2	40.0	±48.6
P009	120.0	±87.8	130.0	±46.0
P010	20.0	±80.3	40.0	±64.1
P011	25.0	±77.7	25.0	±54.5
P012	45.0	±78.4	10.0	±70.0
P013	35.0	±99.1	100.0	±73.1
P014	-20.0	±83.9	50.0	±75.6
P015	70.0	±63.5	50.0	±40.6

**Table 3:** Subject-specific absolute errors in tidal volume estimation. Positive median values indicate that the model predicts a larger tidal volume than the reference measurement. The aggregated row (’All’) represents the overall performance across all 15 subjects.

Subject ID	Tidal Volume Error [l]	
	median	std
All	0.287	±0.202
P001	0.383	±0.124
P002	0.119	±0.044
P003	0.197	±0.107
P004	0.663	±0.225
P005	0.398	±0.171
P006	0.332	±0.126
P007	0.265	±0.074
P008	0.192	±0.099
P009	0.597	±0.154
P010	0.646	±0.115
P011	0.334	±0.160
P012	0.086	±0.129
P013	0.520	±0.259
P014	0.632	±0.161
P015	0.314	±0.155

## Data Availability

Due to the sensitivity of our research data, participants of this study did not agree for their data to be shared publicly, so supporting data is not available.

## Abbreviations

The following abbreviations are used in this manuscript:

ADC	analog-to-digital converter
BCG	ballistocardiography
CW	continuous wave
FMCW	frequency-modulated continuous wave
IQ	in-phase and quadrature
iPPG	imaging photoplethysmography
LDV	laser Doppler vibrometry
MLP	multi-layer perceptron
PCB	printed circuit board
PPG	photoplethysmography
RX	receive
SNR	signal-to-noise ratio
TX	transmit
UHF	ultra-high frequency
